# Current Enzooticity of *Dirofilaria immitis* and *Angiostrongylus vasorum* in Central and Southern Italy

**DOI:** 10.3390/ani15020172

**Published:** 2025-01-10

**Authors:** Donato Traversa, Simone Morelli, Angela Di Cesare, Chiara Astuti, Alessandra Barlaam, Mariasole Colombo, Fabrizia Veronesi, Barbara Paoletti, Raffaella Iorio, Raffaella Maggi, Alessandra Passarelli, Alessia Pede, Linda Rossi, Manuela Diaferia

**Affiliations:** 1 Department of Veterinary Medicine, University of Teramo, 64100 Teramo, Italy; smorelli@unite.it (S.M.); adicesare@unite.it (A.D.C.); castuti@unite.it (C.A.); mcolombo@unite.it (M.C.); bpaoletti@unite.it (B.P.); riorio@unite.it (R.I.); linda.rossi@studenti.unite.it (L.R.); 2 Department of Agricultural Sciences, University of Foggia, 71122 Foggia, Italy; alessandra.barlaam@unifg.it; 3 Department of Veterinary Medicine, University of Perugia, 06126 Perugia, Italy; fabrizia.veronesi@unipg.it (F.V.); manuela.diaferia@unipg.it (M.D.); 4 Freelance Veterinary Practitioner, 00189 Rome, Italy; raffy.maggi@hotmail.it; 5 Clinica Veterinaria Città di Bari, 70125 Bari, Italy; alessandra_pax@hotmail.com; 6 Boehringer Ingelheim Animal Health Italia, 20139 Milan, Italy; alessia.pede@boehringer-ingelheim.com

**Keywords:** *Dirofilaria immitis*, *Angiostrongylus vasorum* dog, Italy, epidemiology

## Abstract

*Dirofilaria immitis* and *Angiostrongylus vasorum* are major parasitic nematodes of dogs. Many environmental and phenological changes have recently modified their geographic patterns in many countries; thus, this study has updated the distribution of *D. immitis* and *A. vasorum* in dog populations of selected regions of Central and Southern Italy. Also, collateral data on other endoparasites affecting the study population have been collected. Blood and fecal samples collected from 2000 dogs were tested using Knott’s test and copromicroscopy (i.e., Baermann’s and fecal flotation tests), respectively. Binomial logistic regression was performed to evaluate statistically significant associations between positivity for *D. immitis* and/or *A. vasorum* and potential risk factors. Overall, 35 (1.7%) and 62 (3.1%) dogs were positive for microfilariae of *D. immitis* and first stage larvae (L1) of *A. vasorum*, respectively, while 3 (0.1%) were co-infected by both nematodes. Microfilariae of *Dirofilaria repens* were found in 148 (7.4%) dogs, while at the flotation, eggs of Ancylostomatidae, *Trichuris vulpis,* and ascarids were found in the feces of 323 (16.5%), 249 (12.4%), and 172 (8.6%), dogs, respectively. Overall, 217 (10.8%) and 44 (2.2%) dogs were positive for eggs of *Capillaria aerophila* and *Capillaria boehmi*. The presence of cardiorespiratory clinical signs or non-specific signs, history of travel, and an age of >4 years old were significantly associated with positivity for *D. immitis*, while *A. vasorum* was significantly recorded in dogs with cardiorespiratory signs, or with a history of mollusk ingestion or permanent outdoor housing. These results confirm that *D. immitis* is enzootic in the investigated regions of Central and Southern Italy, even where it was rare/undetected until recently. Indeed, although some dogs positive for *D. immitis* had a history of travel in enzootic areas, the majority of them were never moved, indicating that they acquired the parasite in the region where they live. Additionally, *A. vasorum* is stably enzootic in the study areas, as also are other extraintestinal nematodes (i.e., *D. repens* and *C. aerophila*) that are more frequently detected today than in the past. A high level of vigilance and routine parasitological screening are necessary, considering the high prevalence of intestinal parasites in owned dogs that are also co-infected by respiratory parasites. The implementation of chemoprevention against *D. immitis* in dogs living in the examined area should be encouraged.

## Simple Summary

*Dirofilaria immitis* and *Angiostrongylus vasorum* are major parasitic nematodes affecting the pulmonary arteries of dogs. In Italy, these two parasites have recently changed their distribution patterns and have been detected in areas where their presence was previously unexpected. The present study has updated available data on the distribution and prevalence of *D. immitis* and *A. vasorum* in canine populations living in selected regions of Central and Southern Italy. The results indicate that both parasites occur stably in the investigated territories and that increased awareness and surveillance methods are of utmost importance.

## 1. Introduction

*Dirofilaria immitis* and *Angiostrongylus vasorum* are major cardiorespiratory parasites of dogs transmitted by intermediate hosts, i.e., mosquitoes and terrestrial gastropods, respectively; paratenic hosts (e.g., frogs) may also be a source of infection with *A. vasorum* [1,2]. The adults of both nematodes reside in the pulmonary arteries of definitive hosts and cause different clinical signs. *Dirofilaria immitis* is responsible for canine heartworm disease, which is a chronic cardiorespiratory illness that can be fatal if not treated [3]. Humans may be accidentally infected by *D. immitis* and suffer from pulmonary infarctions radiologically detectable as “coin lesions” [4]. Dog angiostrongylosis is an unpredictable disease and may present with different clinical presentations, including different combinations of non-specific, cardiorespiratory, neurological, gastrointestinal, and coagulopathy-related clinical signs [5,6,7].

The geographical distribution of *D. immitis* and *A. vasorum* in Europe is influenced by various environmental factors including climate change, the introduction of new invasive vector species (e.g., *Aedes albopictus*, *Aedes koreicus* for *D. immitis,* and *Arion vulgaris* for *A. vasorum*), urbanization, and increased dog movements and relocation [8]. In recent years, a changing distribution and the spread of both nematodes in previously non-enzootic areas have been documented across Europe. In particular, *D. immitis* is establishing itself in central and northeastern regions of Europe where only imported or isolated cases were previously known, and at the same time, *A. vasorum* is constantly reported in European enzootic and non-enzootic territories [1,8].

In Italy, a progressive southward expansion of *D*. *immitis* has been observed in the last decade, and autochthonous foci in previously non-enzootic areas have been documented [9,10]. Similarly, *A. vasorum* has been recently detected in areas where its presence was previously unexpected [11], with a simultaneous rise in cases in enzootic regions, even in co-infections with *D. immitis* [12,13]. Therefore, the present study aimed to update the current knowledge on the infections caused by *D. immitis* and *A. vasorum* in selected regions of Central and Southern Italy where novel information on their occurrence is warranted. The presence of other extraintestinal and intestinal parasites affecting dogs has been also investigated.

## 2. Materials and Methods

### 2.1. Study Design

**Figure 1:**
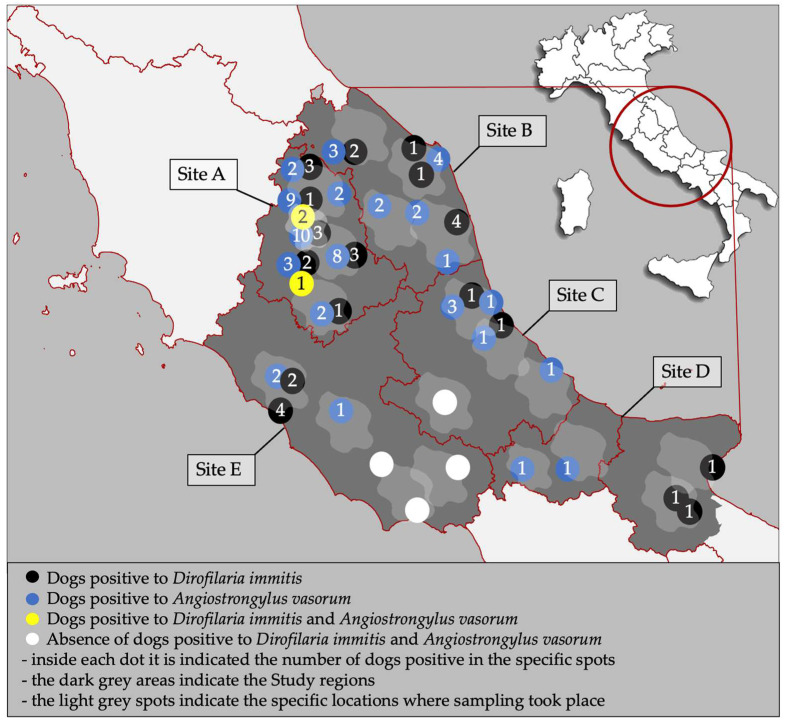
Study sites and distribution of dogs positive for *Dirofilaria immitis* and *Angiostrongylus vasorum* in the present study. Light-grey spots indicate the specific areas of sampling within the regions.

From October 2023 to June 2024, a total of 2000 dogs were selected in regions of Central and Southern Italy, i.e., Umbria (Site A—n. 400), Marche (Site B—n. 400), Abruzzo (Site C—n. 400), Molise and northern Apulia (Site D—n. 366), and Latium (Site E—n. 434) (Figure 1). Dogs were referred to their veterinarians for routine check-up or because they presented clinical signs.

Only dogs fulfilling the following inclusion criteria were included in this study: (i) older than 1 year, (ii) not treated in the previous six months with antiparasitic drugs used for the prevention of dirofilariosis and/or control of angiostrongylosis, and (iii) regular or permanent outdoor access.

For each dog, information on the signalment, history, and presence of clinical signs was recorded. The occurrence of cardiorespiratory, neurological, ocular, non-specific signs and signs possibly related to coagulation disorders was investigated. Individual whole blood and fecal samples were collected for each dog. A signed informed consent was obtained from the owner of/the individual responsible for each dog, and this study was approved by the Ethical Committee of the Department of Veterinary Medicine of the University of Teramo (Prot. No. 34556, 18 October 2023). Detailed demographic data are reported in Table 1.

### 2.2. Laboratory Techniques

Blood samples were examined using Knott’s test for the detection of circulating microfilariae [14]. Fecal samples were subjected to copromicroscopy via Baermann’s test for nematode larvae and to a standard flotation with a 1.350-specific-gravity zinc sulfate solution for the detection of helminth eggs and (oo-)cysts of protozoa [14]. All blood samples that tested positive for microfilariae were subjected to a confirmatory rapid test for the detection of circulating *D. immitis* antigen (Snap4DX—IDEXX Laboratories, Inc., Westbrook, ME, USA).

### 2.3. Statistical Analysis

Statistically significant associations between positivity to *D. immitis*/*A. vasorum* and possible risk factors, i.e., presence of clinical signs, sex, age, gastropod ingestion, history of travel, hunting, cohabitation with other dogs, and housing, were evaluated with binomial logistic regression using the software GraphPad Prism^®^ 10.1.1. Associations were considered significant when the *p*-value (*p*) was ≤0.05. The odds ratio (OR) and the relative 95% confidence interval (95% CI) were calculated. The median age of the dog population studied was 4 years; thus, the dogs were divided into two groups for the statistical analysis on age, i.e., >4 years old and ≤4 years old.

## 3. Results

Out of the 2000 examined dogs, 35 (1.7%) were positive for *D. immitis* microfilariae via Knott’s test. For all of them, the infection was confirmed by the SNAP 4DX test. Upon Baermann’s test, larvae of *A. vasorum* were found in 62 (3.1%) fecal samples [Table 2]. Three dogs in Site A were co-infected with *D. immitis* and *A. vasorum*, of which one was also infected with *Capillaria aerophila* (syn. *Eucoleus aerophilus*) and Ancylostomatidae and another one with ascarids, Ancylostomatidae, and *Trichuris vulpis*. Overall, 22 dogs were co-infected with *D. immitis* and at least one other nematode (11, 6, 0, 2, and 3 in sites A-E, respectively), while 41 dogs were co-infected with *A. vasorum* and at least one other nematode (i.e., 28, 8, 2, 2, and 1 in sites A–E, respectively).

Microfilariae of *Dirofilaria repens* and *Acanthocheilonema reconditum* were found in 148 (7.4%) and 6 dogs, respectively. The most common parasites found via flotation were Ancylostomatidae (16.5%), followed by *T. vulpis* (12.4%) and *C. aerophila* (10.8%) [Table 3].

Infection rates for the individual parasites detected in each study site are shown in Table 2 and Table 3.

Out of the 35 dogs positive for *D. immitis*, 14 (40%) displayed at least one clinical sign, and of them, 9 (25.7%) showed more than one category of clinical signs. Among dogs positive for *A. vasorum*, 26/62 (41.9%) had at least one clinical sign, with 10 (16.1%) suffering from more than one category of clinical signs.

Details on the clinical signs shown by dogs infected with *D. immitis* and/or *A. vasorum* are presented in Table 4.

### Statistical Analysis

Statistically significant associations were found among the presence of cardiorespiratory signs (*p* < 0.001; OR = 4.93; 95% CI = 2.00–12.20), the presence of non-specific signs (*p* = 0.044; OR = 2.41; 95% CI = 1.03–5.67), history of travel to other regions of Italy (*p* = 0.003; OR = 3.47; 95% CI = 1.55–7.79), age of >4 years old (*p* = 0.041; OR = 2.15; 95% CI = 1.03–4.47), and positivity to *D. immitis* [Table 5].

Also, *A. vasorum* infection was significantly associated with the presence of cardiorespiratory signs (*p* < 0.001; OR = 3.51; 95% CI = 1.79–6.90), mollusk ingestion (*p* < 0.001; OR = 4.74; 95% CI = 2.72–8.26), and permanent outdoor housing (*p* = 0.029; OR = 0.50; 95% CI = 0.27–0.93) [Table 5].

## 4. Discussions

The results of the present study show that *D. immitis* is enzootic in the examined areas of Central and Southern Italy, even in regions where its presence was unexpected or undetected in the last decades; at the same time, the stable occurrence of *A. vasorum* in the same areas is confirmed [15,16].

In a recent large-scale survey involving most of the areas examined here (i.e., Sites B–E), *D. immitis* was not found using Knott’s test [16]. However, the parasite was detected in other recent sero-epidemiological investigations carried out in various territories, including Sites A–C and E [16,17,18], and a recent retrospective analysis of serological tests performed by reference diagnostic laboratories showed a steady increase in the prevalence of *D. immitis* in dogs from Central Italy [9]. Thus, there is a factual increased distribution of *D. immitis* in the areas examined here, with regional infection rates higher than in the past in certain regions, e.g., Sites A and E [16,17].

These changes are likely influenced by different factors; among these, dog movements and relocation play a key role. Dogs infected with *D. immitis* may spread the parasite to non-enzootic regions when travelling with their owners, and dogs that are moved from non-enzootic to enzootic areas may become infected and introduce the parasite in territories previously free of it upon their return [19,20]. This is corroborated by the statistically significant correlation between the history of travel and positivity to *D. immitis* obtained here, underlining the impact on the epidemiology of heartworm disease. In this study, 10 dogs that had a history of travel and were positive to *D. immitis* had previously travelled with their owners to endemic regions of Northern Italy for hunting or holidays. However, the remaining dogs positive for *D. immitis* were never moved/relocated, indicating that they acquired the parasite in the region where they live.

Global warming is among the primary drivers impacting the epidemiology of *D. immitis*, as it enhances the development rate of this nematode in their vectors, favoring its spread and expanding distribution in new territories [21,22]. Accordingly, it appears clear that, in Italy, *D. immitis* has expanded its distribution southwards, and it is now enzootic in dog populations throughout the peninsula. This confirms that heartworm disease should no longer be considered limited to Northern Italy, as traditionally thought [2,10,23].

*Angiostrongylus vasorum* is herein confirmed to occur stably in the examined regions, with positivity rates in line with those obtained in the recent past [16]. On the other hand, it should be considered that the positivity for *A. vasorum* could have been underestimated here because a single fecal sample was examined. A higher infection rate would have probably been recorded here if the three consecutive defecations usually required were examined. However, this was unfeasible due to the high number of study dogs.

In the last two decades, *A. vasorum* has spread in the canine populations of Italy and elsewhere, mainly for bridging infections from wild reservoirs to domestic dogs. In particular, conurbation enhances the possibility of contact and/or habitat sharing between foxes, which are the reservoir hosts of *A. vasorum* [24]. Other drivers that have promoted a stable enzooticity of canine angiostrongylosis in several areas are, again, climatic changes, the invasion of exotic snail species, and dog movements [1]. Therefore, *A. vasorum* is now enzootic in canine populations living in central and southern regions of Italy, where it is now a primary pathogen of dogs. This scenario suggests the need to implement awareness and efficacious control strategies against this disease. As canine angiostrongylosis may be unpredictable, severe, and life-threatening, effective control plans should be implemented in enzootic regions.

Co-infections by *D. immitis* and *A. vasorum* have rarely been documented [12,13]. However, the recent establishment of *D. immitis* in Central and Southern Italy, where *A. vasorum* is highly enzootic, can likely cause an increase in mixed infections, as suggested by the records presented here. This also accounts for northern territories, where the two nematodes occur simultaneously [11]. The occurrence of mixed infections may have an important impact in canine practice, and they should be cautiously approached by clinical practitioners due to the unpredictable clinical consequences and serious difficulties in the diagnostic approaches [12].

Several dogs in the present study were diagnosed with *D. immitis* and/or *A. vasorum* during routine examinations and not for the presence of clinical signs. This underlines the crucial role of frequent parasitological analyses in enzootic regions. In fact, both infections may be subtle, with angiostrongylosis being initially subclinical/mild before the onset of severe and sometimes life-threatening clinical signs [25]. Conversely, heartworm disease is typically chronic with gradual appearance of clinical signs, though acute hazardous events such as caval syndrome, thromboembolism, and pneumothorax may occur suddenly, with possible fatal consequences. Thus, the later the diagnosis, the greater the damage (and their eventual irreversibility) [2]. Thus, routine checks for parasites are even more relevant considering the frequent occurrence of co-infections with other extraintestinal and/or intestinal parasites (see Section 3).

Despite the possible occurrence of subclinical or mild infections, any dog presenting with compatible cardiorespiratory signs should be tested for both diseases, as corroborated by the results of the statistical analysis performed here. Among dogs infected with *D. immitis* or *A. vasorum* that showed cardiorespiratory signs, only few (i.e., one and six, respectively) were co-infected by another respiratory parasite, i.e., *C. aerophila*; thus, this statistical result can be considered as not biased by parasitic co-infections. On the whole, the statistical analyses indicated that, in enzootic areas, heartworm disease should be always suspected even if dogs suffer only from non-specific signs, e.g., fatigue/exercise intolerance and weight loss. Regarding the other possible risk factors, the fact that dogs aging >4 years were more likely than younger animals to be infected by *D. immitis* is with all likelihood due to longer exposure to vectors during life; the correlation between the habit of mollusk ingestion and positivity to *A. vasorum* suggests that dogs primarily become infected by eating intermediate rather than paratenic hosts or via other suggested transmission routes [26,27,28]. The positive statistical association between permanent outdoor housing and positivity to *A. vasorum* is due to the increased chances of mollusk ingestion when dogs are not under owner supervision.

*Dirofilaria repens* has been detected here with higher positivity rates than those obtained previously in both the same and other Italian areas [10,16]. Italy is traditionally one of the European countries with the highest prevalence of canine *D. repens* infection [4,21]. Given that subcutaneous dirofilariosis is considered an emerging parasitic zoonosis in Europe, such increased prevalence is of relevance under both veterinary and public health perspectives [29].

Infections by respiratory *Capillaria* spp. were here found with a higher percentage compared to older studies in the same regions [15,16]. This epidemiological scenario suggests, at the same time, the merit of investigating more in depth the clinical occurrence and impact of respiratory capillarioses in the canine populations of Italy and the need to implement control strategies for these erroneously overlooked parasitosis.

According to previous data gathered in Italy, Ancylostomatidae and *T. vulpis* were here the most frequently detected intestinal parasites [16], followed by ascarids; the high rates of infection with intestinal parasites in owned dogs suggest that they should receive more attention in daily veterinary practice. These results indicate that, other than *D. immitis* and *A. vasorum*, common intestinal parasitic infections may go unnoticed during routine veterinary check-ups. This may be due to the often-subclinical nature of intestinal parasitoses mainly in adult dogs [30]. Nevertheless, overlooked infections may (i) produce chronic intestinal diseases in dogs and (ii) increase the chances of environmental contamination with infective stages, enhancing the risk of infection for other dogs and for humans in the case of zoonotic species [31]. Importantly, the high positivity rate of co-infections with respiratory parasites indicates that dogs living in the study regions are at high risk of being infected simultaneously by both extraintestinal and intestinal nematodes, calling for a focused implementation of control methods [4,30].

## 5. Conclusions

In conclusion, the current epidemiological scenario of cardiopulmonary nematodes affecting dogs in Central and Southern Italy (present data; [10]) calls for a higher level of surveillance and control strategies, as canine heartworm disease should be no longer considered rare or occasional in such regions and angiostrongylosis is stably enzootic.

Increased attention should be given to dog relocation, rehoming, and/or transportation, which can easily determine the establishment of these nematodes, especially *D*. *immitis,* in new areas. This is of paramount importance also considering that, very recently, a macrocyclic lactone (ML)-resistant *D. immitis* strain was reported for the first time, in Europe, in a dog living in Italy but arriving from a US region where ML-resistant heartworm strains are present [30]. To date, autochthonous records of resistant strains have never been described in Europe, where ML are still fully efficacious in the chemoprevention of canine dirofilariosis. Nonetheless, a future introduction and expansion of ML-resistant *D. immitis* is not unlikely due to increased travelling of dogs with their owners and dog relocation [19,32]. Heartworm disease control strategies must include diagnostic testing of any travelling dog and appropriate pharmacological prevention. There is merit to consider chemoprophylaxis against *D. immitis* as a standard practice in the areas examined here and no longer considering it as optional. It is worth underlining that MLs contained in formulations labeled for the prevention of *D. immitis* infection are also often effective against the establishment of *A. vasorum* and *D. repens* infections [30]. Such broad-spectrum formulations available on the market are also effective for the treatment of other extraintestinal and intestinal parasites that could be found in co-infections with *D. immitis* and *A. vasorum*. Accordingly, the awareness of both veterinarians and owners and the implementation of specific and epidemiologically based control plans for *Dirofilaria* spp. and *A. vasorum* and other parasites living in sympatry are crucial to safeguard the health of both animals and humans.

## Figures and Tables

**Table 1:** Demographic data of the dogs enrolled in the present study. Site A: Umbria; Site B: Marche; Site C: Abruzzo; Site D: Molise/northern Apulia; Site E: Latium.

	Site A	Site B	Site C	Site D	Site E	Tot
Owned	387	400	383	313	374	1857
Kenneled	13	0	17	53	60	143
Males	231	228	196	180	234	1069
Females	169	172	204	186	173	931
>4 years old	150	137	185	194	187	853
≤4 years old	250	263	215	172	247	1147
Gastropod ingestion	109	58	27	26	64	284
History of travel	50	96	62	44	13	265
Hunting	136	295	212	44	197	883
Contact/Cohabitation with other dogs	251	331	326	266	391	1565
Permanent outdoor housing	149	202	252	151	303	1057

**Table 2:** Number (n) and percentage (%) of dogs testing positive via Knott’s and Baermann’s tests in the different sites examined in this study. Site A: Umbria; Site B: Marche; Site C: Abruzzo; Site D: Molise/northern Apulia; Site E: Latium.

Test	Parasite	Site A n/400 (%)	Site B n/400 (%)	Site C n/400 (%)	Site D n/366 (%)	Site E n/434 (%)	Tot n/2000 (%)
Knott	*Dirofilaria immitis*	16 (4)	8 (2)	2 (0.6)	3 (0.8)	6 (1.7)	35 (1.7)
	*Dirofilaria repens*	69 (17.2)	52 (13)	10 (2.5)	3 (0.8)	14 (3.4)	148 (7.4)
	*Acanthocheilonema reconditum*	-	3 (0.8)	-	-	3 (0.7)	6 (0.3)
Baermann	*Angiostrongylus vasorum*	39 (10.2)	12 (3)	6 (1.5)	2 (0.5)	3 (0.7)	62 (3.1)
	*Strongyloides stercoralis*	13 (3.2)	11 (2.7)	-	-	-	24 (1.2)

**Table 3:** Number (n) and percentage (%) of dogs testing positive via the flotation method in the different sites. Site A: Umbria; Site B: Marche; Site C: Abruzzo; Site D: Molise/northern Apulia; Site E: Latium.

Parasite	Site A n/400 (%)	Site B n/400 (%)	Site C n/400 (%)	Site D n/366 (%)	Site E n/434 (%)	Tot n/2000 (%)
Ascarids	23 (5.7)	64 (16)	28 (7)	12 (3.3)	45 (10.4)	172 (8.6)
Ancylostomatidae	92 (23)	90 (22.5)	59 (14.7)	17 (4.6)	65 (15)	323 (16.5)
*Trichuris vulpis*	99 (24.7)	83 (20.7)	20 (5)	14 (3.8)	33 (7.6)	249 (12.4)
Taeniidae	2 (0.5)	1 (0.2)	4 (1)	-	-	7 (0.3)
*Capillaria aerophila*	62 (15.5)	63 (15.7)	20 (5)	7 (1.9)	65 (15)	217 (10.8)
*Capillaria boehmi*	21 (5.2)	4 (1)	6 (1.5)	-	13 (3.2)	44 (2.2)
*Dipylidium caninum*	-	2 (0.5)	-	-	-	2 (0.1)
*Cystoisospora* spp.	-	5 (1.2)	5 (1.2)	2 (0.5)	7 (1.6)	19 (0.9)
*Giardia* spp.	20 (5)	14 (3.5)	2 (0.5)	-	2 (0.5)	38 (1.9)
*Strongyloides stercoralis*	-	1 (0.25)	-	-	-	1 (0.05)

**Table 4:** Clinical signs displayed by dogs infected by *Dirofilaria immitis* (n. 35 dogs) and by *Angiostrongylus vasorum* (n. 62 dogs).

***Dirofilaria immitis***			
**Category of Clinical Signs**	**n (%)**	**Clinical Sign**	**n (%)**
Cardiorespiratory signs	10 (28.6)	Pale mucous membranes	10 (28.6)
		Dyspnea	2 (5.7)
		Cough	2 (5.7)
Ocular signs	1 (2.85)	Uveitis	1 (2.85)
Non-specific signs	10 (28.6)	Fatigue	5 (14.3)
		Weight loss	5 (14.3)
		Vomiting	1 (2.9)
		Diarrhea	1 (2.9)
No clinical signs	21 (60)	-	-
***Angiostrongylus vasorum***			
**Category of Clinical Signs**	**n (%)**	**Clinical Sign**	**n (%)**
Neurological	2 (3.2)	Seizures	2 (3.2)
Skin lesions	4 (6.4)	Dermatitis	3 (4.8)
		Nodules	1 (1.6)
Cardiorespiratory signs	18 (29)	Dyspnea	5 (8.1)
		Pale mucous membranes	8 (12.9)
		Cough	12 (19.3)
Signs possibly related to coagulation disorders	1 (4.3)	Hemoptysis	1 (4.3)
		Melena	1 (4.3)
		Hematuria	1 (4.3)
Non-specific signs	14 (22.6)	Weight loss	7 (11.3)
		Fatigue	2 (3.2)
		Diarrhea	5 (8.1)
No clinical signs	36 (58.1)	-	

**Table 5:** Results of the binomial logistic regression evaluating statistical associations between potential risk factors and positivity to *Dirofilaria immitis* and to *Angiostrongylus vasorum* in the dogs of the present study.

	*Dirofilaria immitis*			*Angiostrongylus vasorum*		
Factor	*p*	OR	95% CI	*p*	OR	95% CI
Neurological signs	0.994	2.87	0–infinity	0.147	3.67	0.63–21.30
Skin lesions	0.058	1.95	1.32–10.15	0.524	1.44	0.47–4.39
Cardiorespiratory signs	<0.001 *	4.93	2.00–12.20	<0.001 *	3.51	1.79–6.90
Ocular signs	0.989	2.02	0–infinity	0.984	5.79	0–infinity
Signs possibly related to coagulation disorders	0.993	8.43	0–infinity	0.799	1.35	0.13–13.90
Non-specific signs	0.044 *	2.41	1.03–5.67	0.145	1.68	0.84–3.39
Male sex	0.057	2.10	1.00–4.53	0.395	1.27	0.74–2.18
Gastropod ingestion	0.451	1.42	0.57–3.57	<0.001 *	4.74	2.72–8.26
Animal movements (within Italy)	0.003 *	3.47	1.55–7.79	0.092	0.29	0.07–1.22
Hunting	0.975	1.01	0.45–2.30	0.331	1.37	0.73–2.56
Contact/cohabitation with other dogs	0.877	1.07	0.43–2.67	0.439	0.77	0.39–1.50
Permanently outdoor housing	0.527	0.77	0.35–1.72	0.029 *	0.50	0.27–0.93
>4 years old	0.041 *	2.15	1.03–4.47	0.994	1.00	0.58–1.73

* significant result; *p* = *p*-value; OR = odds ratio; CI = confidence interval.

## Data Availability

All the data obtained are included in this manuscript.
